# Precision Global Health – The case of Ebola: a scoping review

**DOI:** 10.7189/jogh.09.010404

**Published:** 2019-06

**Authors:** Nefti-Eboni Bempong, Rafael Ruiz De Castañeda, Stefanie Schütte, Isabelle Bolon, Olivia Keiser, Gérard Escher, Antoine Flahault

**Affiliations:** 1 Institute of Global Health, Faculty of Medicine, University of Geneva, Switzerland; 2 Centre Virchow-Villermé for Public Health Paris- Berlin, Descartes, Université Sorbonne Paris Cité, France; 3 Swiss Federal Institute of Technology (EPFL), Lausanne, Switzerland

## Abstract

**Background:**

The 2014-2016 Ebola outbreak across West Africa was devastating, acting not only as a wake-up call for the global health community, but also as a catalyst for innovative change and global action. Improved infectious disease monitoring is the stepping-stone toward better disease prevention and control efforts, and recent research has revealed the potential of digital technologies to transform the field of global health. This scoping review aimed to identify which digital technologies may improve disease prevention and control, with regard to the 2014-2016 Ebola outbreak in West Africa.

**Methods:**

A search was conducted on PubMed, EBSCOhost and Web of Science, with search dates ranging from 2013 (01/01/2013) – 2017 (13/06/2017). Data was extracted into a summative table and data synthesized through grouping digital technology domains, using narrative and graphical methods.

**Findings:**

The scoping review identified 82 full-text articles, and revealed big data (48%, n = 39) and modeling (26%, n = 21) technologies to be the most utilized within the Ebola outbreak. Digital technologies were mainly used for surveillance purposes (90%, n = 74), and key challenges were related to scalability and misinformation from social media platforms.

**Interpretation:**

Digital technologies demonstrated their potential during the Ebola outbreak through: more rapid diagnostics, more precise predictions and estimations, increased knowledge transfer, and raising situational awareness through mHealth and social media platforms such as Twitter and Weibo. However, better integration into both citizen and health professionals’ communities is necessary to maximise the potential of digital technologies.

While the 2014 Ebola outbreak across West Africa was absolutely devastating, it was also a catalyst for action and innovative solutions in the global health community. The outbreak was the largest recorded in history, accounting for 11 323 deaths and 28 646 confirmed cases, primarily spread across Guinea, Liberia and Sierra Leone, but also affected neighboring countries such as Nigeria, Mali and Senegal [1]. Early estimates projected more than one million cases, mirroring the enormity of the disease burden [2]. Initially an emerging zoonotic viral disease spillover from bats to humans led to introduction of the Ebola virus to humans [3]. Population movement further exacerbated transmission between populations, with mass gatherings for burial services acting as a major hotspot for transmission. The 2014 Ebola outbreak was therefore not only a pivotal event in global health due to its widespread distribution, but also its high fatality rate, which averaged at 50% [3-4].

The public health response to the Ebola outbreak was widely criticized for its lagging response and effectiveness blamed on poor international coordination and collaboration between partners and stakeholders [5-6]. The WHO and their late call for announcing a public health emergency of international concern (PHEIC) was one of the main factors underlying the delayed response. Following the aftermath of the outbreak, many advocated for re-examining the criteria for declaring a PHEIC of the International Health Regulations [5]. While the Ebola outbreak highlighted many fundamental flaws existing within the political and socio-economic environment of the affected countries, the lagging response cannot be attributed to poor coordination and global governance alone. The more traditional diagnostic techniques utilized during the Ebola outbreak, such as the reverse-transcriptase polymerase chain reaction (RT-PCR), antigen-capture enzyme-linked immuno-sorbent assays (ELISA), and serology techniques, contributed to the tipping point toward catastrophe [7]. While these techniques had high sensitivity within the acute phases of the disease, they proved less effective further into infection, and also had longer processing times. The most widely used technique, RT-PCR, was prone to produce a high rate of false-negative results due to improper shipping and storage, and also false-positive results, due to cross contamination [7]. Additionally, these techniques required supporting infrastructure and trained health care professionals, which was often lacking in the most affected countries [6,8].

Lack of core capacities, poor technical assistance, and often-absent operational systems make these health care systems some of the most resource-deprived in the world. While digital innovations have the ability to improve diagnostics, produce more precise alert systems, and increase the efficiency of surveillance, it is important to note that they are only one piece of the complex puzzle [6,8]. Digital health technologies may be defined as digital resources which are used to collect novel personal or environmental data from, and by the populations, including but not confined to: mHealth, social media, remote sensing technologies, GPS, nanotechnology and electronic management databases. An exemplary case of digital technologies is the use of high-throughput genomic sequencing, in which Ebola diagnosis for patients have been achieved in record-breaking time (less than 24 hours) [9]. The emergence of digital technologies has the potential to improve infectious disease surveillance, by providing a timelier, and a more precise response to emergency outbreaks- such as Ebola.

## Aim and research question

This scoping review aimed to provide an overview of the scientific literature focused on digital technologies and the 2014 Ebola outbreak, which may have the potential to strengthen health systems through improved disease monitoring, diagnostic capacity and treatment. The research questions is listed as follows:

What digital technologies were utilized to improve disease prevention and control during the 2014 Ebola outbreak?

## METHODOLOGY

A scoping review aims to ‘form knowledge synthesis that addresses an exploratory research question aimed at mapping key concepts, types of evidence, and gaps in research related to a defined area or field by systematically searching, selecting and synthesizing existing knowledge’, following an established methodological framework [10,11]. This involved the use of a search strategy to identify relevant studies, selection according to inclusion and exclusion criteria, charting the data, collating, summarizing and reporting results [10,11]. The scoping review was not registered, but PRISMA guidelines were followed where applicable [12]. This review aimed to identify the existing digital technologies used to tackle the 2014 Ebola outbreak.

### Search strategy

The search strategy was developed by three authors, and included a broad range of terms related to digital technology and the Ebola outbreak, which consisted of a combination of free text and MeSH terms, in accordance with the PRISMA guidelines [12] (see Table S1 in **Online Supplementary Document**). Digital technology-related search terms were identified through key-terms of a preliminary literature review (see **Online Supplementary Document**), while Ebola–related search terms were identified using the MeSH terms from the National Library of Medicine MeSH database and cataloged synonyms. Technology-related and Ebola-related key terms were combined using Boolean operators, for example: [(Ebola) AND (Technology) OR (Big data) OR (Social media) OR (mHealth)], to identify relevant literature related to the use of digital technologies with regard to the 2014 Ebola outbreak. Using identified search terms the following was conducted:

- Using pre-existing key words and index terms; a search was conducted on medical, nursing, psychological and social science databases.
- Analysis of keywords in title, abstract and index terms.
- Search reference lists of identified material to identify further material of relevance.

To ensure a comprehensive review of the literature, the following databases reporting quantitative and qualitative studies were included in the review: PubMed, Web of Science and EBSCOhost. Additional literature was identified using snowball methodology and hand searching previously identified publications.

### Study selection; inclusion and exclusion criteria

The review considered any empirical studies that discussed the utilization of digital technologies in improving disease prevention and control, with specific reference to the 2014 Ebola outbreak. It considered peer-reviewed articles (including original quantitative and qualitative studies), but also systematic reviews, editorials, viewpoints and letters (see **Table 1**). Text had to be published in the English or French language between 2013 (01/01/2013) and 2017 (13/06/2017), to follow the timeline of the Ebola outbreak. There were no restrictions with regard to geographic location, population or study design. The review excluded duplicate articles or studies with no explicit focus on digital technologies linked to the 2014 Ebola outbreak.

**Table 1:** Overview of study design included in scoping review (n = 82)

Characteristic	Number (n)	Percentage (%)
Descriptive (Cross-sectional and analytical)	**27**	**33**
Modeling (Spatiotemporal analysis, computer modeling, real-time modeling, simulation study)	**22**	**27**
Experimental (Experimental and before and after)	**5**	**6**
Longitudinal (Longitudinal and cohort)	**2**	**2**
Ethnography	**10**	**12**
Content analysis	**8**	**10**
Other (Text mining, retrospective review, case studies, viewpoints, discourse analysis)	**8**	**10**

### Data collection, extraction and synthesis

Two reviewers assessed inclusion and exclusion criteria of titles and abstracts for relevance. Once relevant studies were identified, further appraisal of full text papers was conducted independently, and relevant information was integrated into a descriptive summative table, which focused on: author (s), publication date, journal, study site/area, digital technology/device, function, study design, target population, health indicator (ie, Hard indicators: incidence and disease distribution and soft indicators: health awareness) and challenges. Citations were managed using EndNote software. Data was synthesized using graphical and narrative methods, with the emergence of themes by frequency of use of the application. Furthermore an author’s affiliation network was created to map the existing research, using JavaScript software. The graph was produced by the addition of an edge between the first author and links to each of the other authors, with number of collaborations corresponding to link density.

## RESULTS

**Figure 1:**
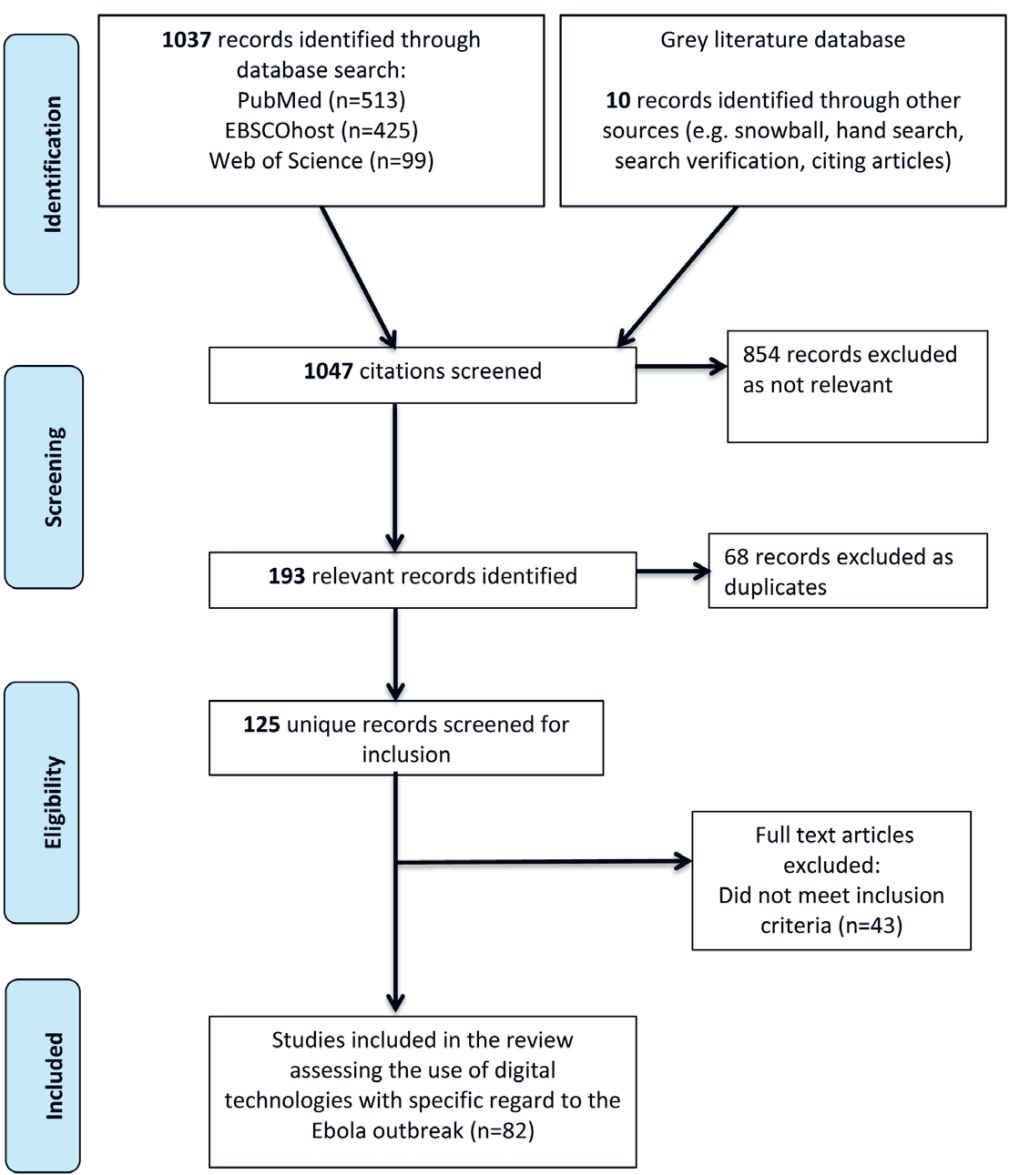
Process of study selection.

**Figure 2:**
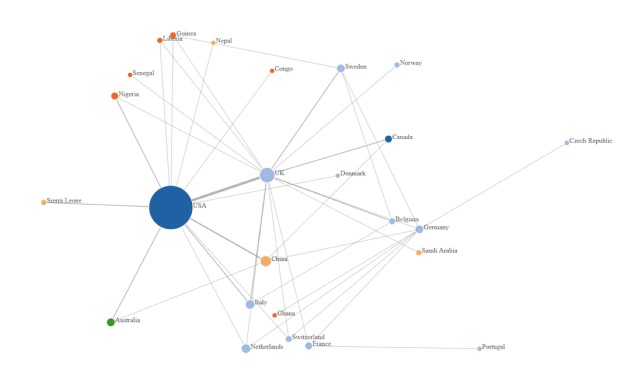
Authors’ affiliation network.

A total of 1047 titles and abstracts were screened, of which 193 were identified as relevant studies. Of the relevant studies 68 were excluded as duplicate studies, and 43 did not meet the inclusion criteria. Therefore, a total of 82 studies were included in the final review (**Figure 1** and **Figure 2**) [9,13-94]. Studies included in the review uncovered digital technology domains related to the most recent Ebola outbreak. The review identified technologies that were utilized throughout the 2014 Ebola outbreak, with the potential to improve disease prevention and control, for a more precise outbreak response. Five main themes emerged from the 82 studies identified within the review, namely big data, mHealth, modeling, novel technologies and remote-sensing technologies (**Table 2**).

**Table 2:** Identified technology domains

Digital technology domain	Description	Specific description within the context of this study	Number (n)	Percentage (%)	References for analyzed papers
Big data	A term describing the storage and analysis of large and or complex data sets using a series of techniques including, but not limited to: cloud computing, non-relational databases, natural language processing and machine learning [95].	Mainly included through the use of big data analytics of social media platforms (eg, Twitter, Facebook, YouTube), MOOCs, and web-based surveillance (HealthMap, Grippenet.ch).	39	48	[13-51]
Modeling	Models involve assumption, abstraction and simplification, of complex disease-associated dynamics [96].	This review encompassed modeling as computer/software assisted modeling, primarily referring to mathematical (Monte-Carlo, Bayesian), computational, spatial-temporal or real-time modeling.	21	26	[52-72]
mHealth	Medical and public health practices supported by mobile devices, such as mobile phones, patient monitoring devices, personal digital assistants, and other wireless devices [97].	Mobile phone devices, cell-phone data generation and its associated functions including: GPS, SMS, voice system and tutorial applications.	12	15	[73-84]
Novel technologies and devices	Case-specific technologies produced or updated, to specifically track and monitor the outbreak, considered “interestingly new or unusual” [98].	Nanotechnologies using nano-magnetic materials and methods were sub-categorised under novel technologies, among other case-specific technologies and devices.	9	10	[9,85-93]
Remote-sensing technologies	Technologies with the ability to identify observe and measure an object without coming into direct contact with it [99].	Remote-sensing technologies under the parameters of satellite telemetry, satellite imagery or the use of drones.	1	1	[94]

### Big data

39 studies (47%) found in the review focused on big data, under the parameters of big data analytics and web-based surveillance, mostly used for the purposes of health communication and monitoring levels of awareness regarding Ebola. Big data analytics were applied to major social media platforms, such as Twitter, Facebook and YouTube, but also country-specific platforms such as China’s Weibo. Big data was also present in the form of online surveillance-mapping tools, such as the HealthMap tool, and was also extended to zoonotic mapping portals.

### Modeling

21 studies (26%) were identified with reference to modeling within this review. Most modeling was mirrored in computer or software-assisted mathematical modeling, including but not confined to: Bayesian probabilities and inferences, Monte-Carlo simulations, but also spatial-temporal modeling combined with GPS/GIS data.

### mHealth

The review identified 12 studies (15%) that utilized cell-phone technology linked to the Ebola outbreak. mHealth was mostly adapted for learning purposes, and also for the dissemination of information. The GPS function of mobile devices also enabled mHealth technologies to be used for the purposes of contact tracing.

### Novel technologies

The review also identified nine Ebola-specific novel technologies (11%), presented as novel, as they were specifically adapted to help tackle the 2014 Ebola outbreak. These technologies were utilized with the aims of improving working conditions for health workers, strengthening monitoring, and reducing the spread of diseases. It is important to note that the presence of biotechnologies was strong under the umbrella term of novel technologies, referring primarily to nanotechnologies.

### Remote-sensing technologies

The review identified one study (1%), under the remote-sensing domain via the use of satellite technology. High-resolution satellite imagery was utilized to better visualize spatial-temporal targets for disease.

Overall, big data technologies (47%) and modeling (26%) were the most utilized digital technologies throughout the Ebola outbreak (**Table 2**). Digital technologies identified within this scoping review were mainly utilized for surveillance (90%), with very low use observed for both diagnostics (8%) and treatment (2%). Some of the main barriers and challenges identified in the application of technologies are listed below (**Table 3**), such as technical standards and quality, and also lack of health information technology infrastructure. In the case of Ebola it became evident that the highest research output (authors of articles) originated from the USA, who mostly collaborated with the UK, Italy and China.

**Table 3:** Barriers identified in the application of technologies during the Ebola outbreak.

Barrier	Related issues
Digital divide	• Unbalanced media coverage • Inconsistent cell-phone and Wi-Fi coverage • Internet connectivity • No integration of social media use in curriculum
Technical standards and data quality	• Underreporting cases • Poor baseline data • Production of false-positives • Data volume and complexity
Ethics, Law, Social Science, Anthropology (ELSA)	• Literacy gap between males and females
Healthcare system and incentives	• Lack of trained staff • Lack of training and integration • Intervention scalability • Missing health records • Missing exposure data
Confidence and trust	•Trustworthy news outlets • Misinformation

## DISCUSSION

Findings from the review identified five main themes on the use of digital technologies in the Ebola outbreak. Among the studies included in the review, Big data and modeling technologies were found to be the most utilized (**Table 2**). Social media platforms such as Twitter and Facebook lent themselves as seamless tools for increasing situational awareness, and also facilitating the knowledge transfer regarding Ebola [13-15]. Additionally, the review identified the USA as the largest hub for digital innovation, mirrored by the highest research output in academia (**Figure 2**).

Big data [13-52] was the most represented in the literature, an exemplary case being the Twitter-based “@KickEbolaOut” campaign in Nigeria, which was founded in community mobilization and action. The campaign was launched by medical students in hopes of increasing overall knowledge about Ebola transmission, and also aimed to reduce community perceptions and stigma related to Ebola [17]. Big data was also heavily present in the form of online surveillance-mapping tools, including the HealthMap tool and the Surveillance and Outbreak Response Management System (SORMAS) [18,19]. HealthMap is notorious for announcing the first public notification regarding Ebola in March of 2014, 5 months before the WHO declared Ebola as an PHEIC [20]. The HealthMap tool exemplifies how real-time digital intelligence has the potential to improve early detection, granting a timely response to public health emergencies. Findings from this scoping review thus indicate that big data does not only have the ability to map distribution, but may also be a valuable tool for gaining insight into health seeking behaviors from the general public [13-15,17,22]. The ability to easily update and add to online data sources, allows web-based systems to generate real-time data at a quicker rate than those achieved by more classical epidemiological tools.

Applied models were also greatly used throughout the outbreak, as they enable prediction of transmissibility, spreading/risk patterns, production of an epidemic forecast, and also effectiveness of various interventions that may or may not be implemented [52]. The Spatiotemporal Epidemiological Modeler (STEM) is a web-based open source tool, which functions through a combination of mathematical modeling and geospatial epidemiological modeling, utilizing the geographic information system [54]. The STEM was based on the mathematical *SIR* model, where *S –* equaled the number of those susceptible, *I –* the number of infected patients and *R –* the number of recorded or immune individuals. Although modeling may be considered rather classical, the combination of mathematical equations, GIS/GPS functions, computer and/or software assistance and visualization technologies, introduces an innovative aspect, ideal for application in the global health sphere.

Cell phone technology, or mHealth, has been a big theme in low and middle-income countries, most likely due to increased access and higher mobile phone coverage and usage [82,84]. Cell phone technology was not only able to produce real-time visualization of social networks via mobile mapping, but also tracked population movement and disseminated information [75,77]. mHealth was also adapted for learning via an app for predicting prognosis named ‘Predictor Pipeline’, and tutorial applications regarding Ebola transmission and common symptoms [73]. A study in Nigeria revealed an 11% statistically significant improvement in average knowledge level regarding Ebola, pre- and post-intervention [73]. The increased accessibility and user-friendly nature of mobile phones thus qualifies mHealth technologies as an optimal candidate to improve disease monitoring. Throughout the Ebola outbreak, a participatory approach was adopted for data collection, mirrored by the use of reporting cases via SMS and updating web-based surveillance systems in ‘real-time’ [78]. Studies confirmed improvements in reporting, reaching 85% of suspected cases on a daily basis following introduction of mHealth technologies [75]. A noteworthy study reported women at reduced odds of making a positive call (a call which presents listed symptoms of a suspected case) prior to the intervention of mHealth, but integration of mHealth technologies into the community increased user acceptance, and was also reported to empower women [75].

The review also identified Ebola-specific technologies, such as the wearable, wireless ‘Band-Aid’ sensor with personalized analytics, which used wireless transmission to send patient specific alerts to health care workers, eliminating the need for the physical presence of health care workers to conduct check-ups [88]. Additionally, the use of nanotechnologies has enabled diagnostics to shift from more traditional techniques such as cell culture and immunological assays, to more molecular based diagnostics [86,89,90]. The Oxford Nanopore MinION device is in the form of a universal serial bus (USB), and its function is governed by frequent electrical current measurements, as a single strand of DNA passes through the protein nanopore. Real time genomic sequencing allows for longer molecules of DNA to be read (50kb or longer), and despite higher error rates, still has the ability to determine accurate genotypes at much faster processing times [9].

The scoping review revealed that digital technologies might also be utilized at different stages of the Ebola outbreak, ranging from diagnostics and training of health care workers, to treatment management and follow-up. Within the parameters of diagnosis, digital technologies were able to accelerate processing times, by stepping away from more traditional techniques to rapid, real-time diagnostics. A particularly notable example was the use of a palm-sized point of care device, which successfully achieved diagnosis within 37 minutes, while also being practical for use [83]. The introduction of novel nanotechnologies throughout the Ebola outbreak also contributed to improved diagnostics, primarily through minimising the need for laboratory-specific equipment and supporting infrastructure [89].

Additionally, the use of digital technologies also contributed to strengthening learning systems for communities of affected areas. The Ebuddi prototype tested in Liberia utilized digital technology to improve the training process through a graphical user interface, with adapted animation and language settings [80]. Initially Ebuddi was launched on PCs, however it quickly became apparent users were unfamiliar with the use of computers, which thus required technology to be tailored to users, and also indicated technological leapfrogging [80]. The latter concept refers to moving rapidly to the use of modern technologies ie, touch-screen phones, without familiarisation of intermediate steps ie, non-touch screen devices. However, learning systems were not confined to the creation of a learning system for affected communities, but also aimed to educate on a more global level through the use of Massive Open Online Courses (MOOCs) [38]. An Ebola-specific MOOC was created on an online portal to educate the general population on the cause, transmission route and symptoms of Ebola [38]. The ability for MOOCs to reach many people very rapidly is highly advantageous for the dissemination of information, particularly in the context of an infectious epidemic.

However, it is also important to consider that the introduction of technology throughout the Ebola crisis had associated costs and challenges. The ethical implications regarding public disclosure of names was perhaps one of the greatest issues encountered, as this violation of privacy would often result in vilification of said individuals by the media [23]. Identified individuals were therefore less likely to seek treatment, and were often stigmatised and ostracised by their communities [94]. These social effects also had an incidental effect on data quality and management of open data mapping portals, as identified cases often refused to come forth with any personal information, reducing the accuracy of the information gathered [23,26,30]. The vilification of affected individuals was exacerbated by circulating beliefs among the local communities, who believed Ebola to be a ‘satanic and bewitched disease’ [94]. Misinformation, particularly on social media platforms, was a huge challenge, as false and incorrect information was often shared [30]. These challenges highlighted the need to continue strengthening the learning systems, and trust within affected areas.

Studies also noted increased use of technologies among males when compared to females, which could most likely be due to males having the dominant role in the household, and also having a higher literacy rate [28]. The 4A perspective, namely referring to awareness, access, attitudes and applications, hypothesizes the underlying reasons may be attributed to socio-cultural conditioning [26]. This, for instance, may refer to reduced access to education for females, limited free time due to domestic responsibilities and being primary care takers, as well as financial and constitutional constraints [26]. Misinformation was another obstacle, particularly for the use of social media platforms, in which a study analyzing text on Twitter, found 58.9% of Tweets to contain medical misinformation [26]. Additionally, a study that focused on reporting cases via mHealth, found that 82% of reported deaths did not meet the case definition [55]. This again places emphasis on the need to better regulate big data platforms, to prevent circulating misinformation.

It is important to note that there were also some methodological limitations concerning the scoping review. The first acknowledges that only three databases were used to search for literature, thus the existing evidence base may not have been adequately captured. Additionally, bias, specifically publication bias, may have been introduced into the study, as not all relevant studies may have been published in the time frame proposed, and not all sources of data were included in this review. This suggests that unsuccessful applications may have been less likely to be published. Lastly, synthesis of both quantitative and qualitative findings was a challenge, as statistical analysis could not be conducted, resulting in a more narrative and graphical summary of findings. Beyond the case of Ebola, the challenge with digital technologies within health remains with scaling up innovations, such as those uncovered in the present review. Despite successful pilot interventions, the experimental nature of digital technologies keeps them confined within context, and as a result – successful local pilots are rarely adopted at regional or national level. However, despite the challenges associated with digital technologies, they still offer great potential in improving emergency response and positively shaping health outcomes. Social media platforms offer great potential in acting as both tools for communication and situational awareness, but also gauging health-seeking behaviors. The low level of research published in utilizing technologies for diagnostic and treatment purposes also introduces rationale for innovative solutions within those functions.

## CONCLUSION

This review found that the Ebola outbreak signaled the shift from the old to the new, where digital technologies for more precise predictions and estimations, were used to respond to, and tackle the outbreak. The main advantage of using technologies is the speed and precision at which tasks can be executed and completed, rooted within a participatory approach. However, challenges still remain – such as privacy and security, willingness to share information and studies suggesting males as the predominant users of technology. Despite this, digital technologies still offer great potential in coordinating a more precise response to outbreaks, and also shaping health outcomes. The realm of digital technology is constantly expanding, and as it evolves, so must we.

## Additional material

Online Supplementary Document
