# Supplementary material for: Precision Global Health – The case of Ebola: a scoping review
Source: J Glob Health. 2019 Jan 22;9(1):010404. doi: 10.7189/jogh.09.010404 (PMC6344070; doi:10.7189/jogh.09.010404)
Supplement: Online Supplementary Document [file jogh-09-010404-s001.pdf]

J Glob Health 2019; 9: 010404

Table S1: Search Strategy Syntax for PubMed

**Database**	**Search Syntax**
PubMed	("Ebola"[Title/Abstract] OR "Ebola virus disease"[Title/Abstract] OR "EVD"[Title/Abstract] OR "EBOV"[Title/Abstract] OR "Zaire"[Title/Abstract] OR "Viral disease"[Title/Abstract]) AND ("Digital"[Title/Abstract] OR "Technology"[Title/Abstract] OR "Precision medicine"[Title/Abstract] OR "Biosensor"[Title/Abstract] OR "Sensors"[Title/Abstract] OR "Bio-surveillance"[Title/Abstract] OR "Intelligent surveillance"[Title/Abstract] OR "Participatory surveillance"[Title/Abstract] OR "Genomic epidemiology"[Title/Abstract] OR "Genomic sequencing"[Title/Abstract] OR "Pathogen genomics"[Title/Abstract] OR "Big data"[Title/Abstract] OR "Data storage"[Title/Abstract] OR "Data science"[Title/Abstract] OR "Information processing"[Title/Abstract] OR "Blockchain"[Title/Abstract] OR "Social media"[Title/Abstract] OR "Twitter"[Title/Abstract] OR "Facebook"[Title/Abstract] OR "Instagram"[Title/Abstract] OR "Flicker"[Title/Abstract] OR "YouTube"[Title/Abstract] OR "Wikipedia"[Title/Abstract] OR "Telemedicine"[Title/Abstract] OR "Robotics"[Title/Abstract] OR "Machine learning"[Title/Abstract] OR "Modelling"[Title/Abstract] OR "Mathematical modelling"[Title/Abstract] OR "Spatiotemporal modelling"[Title/Abstract] OR "Mapping"[Title/Abstract] OR "mHealth"[Title/Abstract] OR "Mobilephone"[Title/Abstract] OR "Smartphone"[Title/Abstract] OR "Cellphone"[Title/Abstract] OR "Phone"[Title/Abstract] OR "Cell phone technology"[Title/Abstract] OR "Mobile data"[Title/Abstract] OR "Mobile application"[Title/Abstract] OR "Devices"[Title/Abstract] OR "Connected device"[Title/Abstract] OR "Internet"[Title/Abstract] OR "Web-based"[Title/Abstract] OR "Internet-based"[Title/Abstract] OR "Web-database"[Title/Abstract] OR "Cloud"[Title/Abstract] OR "Cloud-based"[Title/Abstract] OR "eHealth"[Title/Abstract] OR "E-learning"[Title/Abstract] OR "Game-based learning"[Title/Abstract] OR "Augmented reality"[Title/Abstract] OR "Massive Online Open Courses"[Title/Abstract] OR "MOOC"[Title/Abstract] OR "Virtual

	learning"[Title/Abstract] OR "Virtual reality"[Title/Abstract] OR "Online learning"[Title/Abstract] OR "Gaming technology"[Title/Abstract] OR "Serious game"[Title/Abstract] OR "Crowd sourcing"[Title/Abstract] OR "Citizen Science"[Title/Abstract] OR "Connected device"[Title/Abstract] OR "Remote-sensing technology"[Title/Abstract] OR "Satellite"[Title/Abstract] OR "GPS"[Title/Abstract] OR "Global Positioning System"[Title/Abstract] OR "Geographic Information System"[Title/Abstract] OR "GIS"[Title/Abstract] OR "Spatial"[Title/Abstract] OR "Participatory"[Title/Abstract] OR "Sensor"[Title/Abstract] OR "App"[Title/Abstract] OR "Artificial intelligence"[Title/Abstract] OR "Tracking"[Title/Abstract] OR "Mapping"[Title/Abstract] OR "Biogeography"[Title/Abstract] OR "Biomarkers"[Title/Abstract] OR "Disease mapping"[Title/Abstract])

Authors affiliation network:
https://s3.amazonaws.com/salathegroup-static/misc/mohanty/author-graphs/ebola.html
